# Chronic kidney disease in congenital heart disease patients: a narrative review of evidence

**DOI:** 10.1186/s40697-015-0063-8

**Published:** 2015-08-11

**Authors:** Catherine Morgan, Mohammed Al-Aklabi, Gonzalo Garcia Guerra

**Affiliations:** Division of Nephrology, Department of Pediatrics, 4-557 Edmonton Clinic Health Academy, 11405 - 87 Avenue, Edmonton, AB T6G 1C9 Canada; Division of Cardiac Surgery, Department of Medicine, 4A7.C Mazankowski Heart Institute, 8440 - 112 Street, Edmonton, AB T6G 2B7 Canada; Division of Pediatric Critical Care, Department of Pediatrics, 4-548 Edmonton Clinic Health Academy, 11405 - 87 Avenue, Edmonton, AB T6G 1C9 Canada

**Keywords:** Chronic kidney disease, Congenital heart disease, Cardiac surgery

## Abstract

**Purpose of review:**

Patients with congenital heart disease have a number of risk factors for the development of chronic kidney disease (CKD). It is well known that CKD has a large negative impact on health outcomes. It is important therefore to consider that patients with congenital heart disease represent a population in whom long-term primary and secondary prevention strategies to reduce CKD occurrence and progression could be instituted and significantly change outcomes. There are currently no clear guidelines for clinicians in terms of renal assessment in the long-term follow up of patients with congenital heart disease. Consolidation of knowledge is critical for generating such guidelines, and hence is the purpose of this view. This review will summarize current knowledge related to CKD in patients with congenital heart disease, to highlight important work that has been done to date and set the stage for further investigation, development of prevention strategies, and re-evaluation of appropriate renal follow-up in patients with congenital heart disease.

**Sources of information:**

The literature search was conducted using PubMed and Google Scholar.

**Findings:**

Current epidemiological evidence suggests that CKD occurs in patients with congenital heart disease at a higher frequency than the general population and is detectable early in follow-up (i.e. during childhood). Best evidence suggests that approximately 30 to 50 % of adult patients with congenital heart disease have significantly impaired renal function. The risk of CKD is higher with cyanotic congenital heart disease but it is also present with non-cyanotic congenital heart disease. Although significant knowledge gaps exist, the sum of the data suggests that patients with congenital heart disease should be followed from an early age for the development of CKD.

**Implications:**

There is an opportunity to mitigate CKD progression and negative renal outcomes by instituting interventions such as stringent blood pressure control and reduction of proteinuria. There is a need to invest time, thought and money to fill existing knowledge gaps to improve health outcomes in this population. This review should serve as an impetus for generation of follow-up guidelines of kidney health evaluation in patients with congenital heart disease.

## Abrégé

**Objet de l’étude:**

Les patients atteints de cardiopathie congénitale présentent un certain nombre de facteurs de risque de développer une insuffisance rénale chronique (IRC). On sait que l’IRC comporte de nombreux effets négatifs sur l’état de santé. Les patients atteints de cardiopathie congénitale formeraient donc une population chez qui la mise en place de stratégies de prévention primaires et secondaires en vue de réduire la fréquence et la progression de l’IRC pourrait améliorer considérablement les résultats. Il n’existe actuellement, pour les cliniciens, aucune ligne directrice claire régissant l’évaluation de la fonction rénale dans le cadre du suivi à long terme des patients atteints de cardiopathie congénitale. La mise en commun des connaissances est cruciale pour l’élaboration de telles lignes directrices, d’où l’objet de la présente étude. Celle-ci présentera une synthèse des connaissances actuelles relatives à l’IRC chez les patients atteints de cardiopathie congénitale, afin de souligner le travail important effectué jusqu’ici et de préparer le terrain pour des recherches approfondies, d’élaborer des stratégies de prévention, ainsi que de réévaluer le suivi approprié de la fonction rénale chez les patients atteints de cardiopathie congénitale.

**Sources d’information:**

La recherche documentaire a été effectuée à l’aise de PubMed et Google Scholar.

**Conclusion:**

Les signes épidémiologiques actuels suggèrent que l’IRC se présente chez les patients atteints de cardiopathie congénitale plus fréquemment que chez la population en général et qu’elle peut être détectée de manière précoce (c.-à-d. durant l’enfance). Les données indiquent qu’environ 30 à 50 % des patients adultes atteints de cardiopathie congénitale subissent une détérioration de la fonction rénale. Les risques de développer une IRC sont accrus chez les patients atteints d’une cardiopathie cyanogène congénitale, bien qu’ils existent aussi dans les cas de cardiopathie congénitale non cyanogène. Bien qu’il y ait des lacunes considérables dans les connaissances, la somme des données suggère que les patients atteints de cardiopathie congénitale devraient faire l’objet d’un suivi dès un jeune âge afin de déceler l’apparition de l’IRC.

**Répercussions:**

Il serait possible d’atténuer la progression et les effets négatifs sur la fonction rénale en instituant des interventions telles qu’un contrôle rigoureux de la pression artérielle, ainsi que la réduction de la protéinurie. Un investissement en matière de temps, de réflexion et d’argent serait nécessaire afin de combler les lacunes dans les connaissances, et ainsi améliorer les effets sur la santé de la population en question. La présente étude devrait motiver l’élaboration de lignes directrices pour le suivi de la fonction rénale chez les patients atteints de cardiopathie congénitale.

## What was known before

Several basic science, physiological and epidemiological studies report on the development of CKD in patients in congenital heart disease.

## What this review adds

This review consolidates the current knowledge regarding CKD in patients with congenital heart disease, and places findings from different lines of investigation within a larger context, while making future research and clinical care needs clear.

## Background

With advances in care, children undergoing complex cardiac repairs are surviving more frequently, resulting in a markedly increasing number of adults with congenital heart disease [1, 2]. It is important to think about the impact that these intensive interventions have on organ systems, including the kidney. The kidney is at high risk of long-term negative impact, given the pathophysiological changes that occur in the context of congenital heart disease, surgical intervention and cardiopulmonary bypass, post-operative critical care and recurrent exposure to potential renal insults. Patients with congenital heart disease use substantial healthcare resources, and not just during the time of cardiac repair, but also as surviving adults with congenital heart disease [3]. Chronic kidney disease (CKD) also causes significant personal and economical health care burden and is associated with worse long term outcome, quality of life and well being in the general population [4]. Hence CKD in patients with congenital heart disease has potential for synergistic negative impact.

Although there are a significant number of gaps in the knowledge related to the renal outcomes of children with congenital heart disease and children who have had cardiac surgery, current evidence demonstrates CKD as an increasingly prevalent and important problem in these patients. Given the potential to mitigate CKD development and progression in many different populations, with universally accepted interventions, clinicians, researchers, and policy makers should be interested in this problem from both an economical and patient-centered outcome point of view. This review will consolidate current knowledge related to CKD in patients with congenital heart disease, to highlight important work that has been done and set the stage for further investigation, development of prevention strategies, and re-evaluation of appropriate renal follow-up in children with congenital heart disease and cardiac surgery.

## Review

### Pathophysiology in congenital heart disease can lead to long-term changes in kidney structure and function

**Fig. 1:**
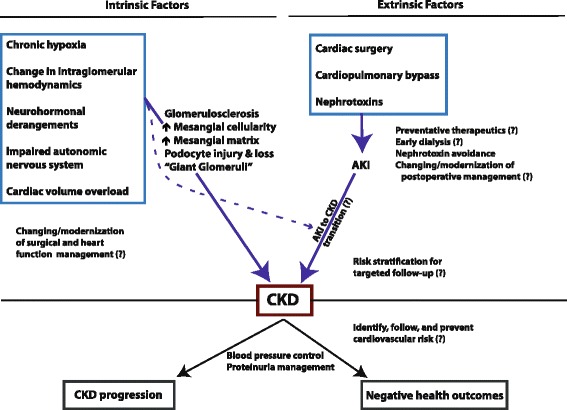
Knowledge synthesis of chronic kidney disease in patients with congenital heart disease; risk factors, disease mechanisms, interventions, and outcomes. “?” indicates a knowledge gap in research or clinical care needs

Children with congenital heart disease have a number of risk factors for potential development of CKD later in life, including pathophysiological changes related to a structurally abnormal heart and circulation. These may include polycythemia, cyanosis and chronic hypoxia, changes in renal blood flow and intraglomerular hemodynamics, and derangements in neurohormonal activation (Fig. 1).

In cyanotic congenital heart disease, chronic hypoxia stimulates erythrocytosis through the stimulation of erythropoeitin, resulting in increase blood viscosity [5–7]. Several decades ago, experimental increase of the hematocrit with resulting hyperviscosity was shown to influence renal hemodynamics, with glomerular hyperfiltration being a predominant feature [8]; hyperviscosity could lead to changes in glomerular arteriolar resistance, hydraulic pressure across the glomerulus, and increased filtration fraction. In a small study by Passwell et al. in 1976, children with cyanotic heart disease were shown to have decreased glomerular filtration rate (GFR) (measured by 24 h urine creatinine clearance) that increased to normal range within 3 months after correction of the heart lesion; the reduced GFR prior to complete surgical correction was associated with high hematocrit [9]. Although Burlet et al. demonstrated normal pre-operative GFR by inulin clearance in 18 children with stable cyanotic congenital heart disease, renal plasma flow (and renal blood flow) was reduced with a resulting increase in filtration fraction [10]; the investigators explanation for this phenomenon was hyperviscosity induced increase in efferent arteriolar tone with resulting glomerular hypertension and rise in effective filtration pressure. In adult patients with a history of Tetralogy of Fallot repair in childhood, assessed at a mean of 20 years after repair (range 18-23), increasing hematocrit was tightly correlated with increasing filtration fraction [11]. Of critical importance is the knowledge that high glomerular hydrostatic pressure and increased filtration fraction can lead to chronic nephropathological changes including glomerulosclerosis [12, 13].

In 1960, Spear described “giant glomeruli” in adult patients with cyanotic congenital heart disease [14], the pathogenesis of which was unexplained at that time. More recently, glomeruli of adults with cyanotic congenital heart disease have been characterized by dilated hilar arterioles, an increase in glomerular capillary diameter with red cell engorgement and glomerular enlargement [15]. The pathogenic mechanism behind these changes is thought to be due to stimulation of intraglomerular nitric oxide release though increased endothelial shear stress, which accompanies increased whole blood viscosity [15].

Non-vascular glomerular abnormalities also suggest additional pathogenic mechanisms in cyanotic congenital heart disease. Increased juxtaglomerular and mesangial cellularity and increased mesangial matrix has been demonstrated, with the presence of a left to right shunt being a major contributing factor [15, 16]. Megakaryocytes normally enter the systemic venous bed from the bone marrow, and make their way to the lungs where they shed platelets. In the presence of a left-to-right shunt, megakaryocytes could enter the systemic arterial circulation and fortuitously lodge in the glomerular capillaries where they release high concentrations of mitogens/chemokines including platelet derived growth factor and transforming growth factor beta [15].

Chronic local tissue hypoxia has been shown to have an important role in CKD development in a number of disease states, including diabetes, hypertension, and cyclosporine toxicity [17]. It is quite plausible that chronic hypoxia associated with congenital heart disease could have an equal role in the initiation and development of progressive renal disease in this population. There is evidence of a mitogenic and fibrogenic role for hypoxia in several cell systems, including renal tubular epithelium and glomerular mesangial cells [16, 18]. Hypoxia induces a significant increase in the mRNA levels of extracellular matrix proteins including type IV collagen, fibronectin, and laminin in cultured rat mesangial cells, human proximal tubular epithelial cells and human cortical interstitial fibroblasts [16, 19]. The signaling pathway through which hypoxia increases cell proliferations and matrix expression in the kidney is thought to involve transforming growth factor beta, osteopontin, nitric oxide, and p38 mitogen-activated protein kinase [16]. Although no studies have evaluated the time course for the development of hypoxia-induced changes in congenital heart disease, animal models demonstrate that renal tubular structure is abnormal and interstitial fibrosis is present after a relatively short period of tissue hypoxia (28 days in rats) [20].

Neurohormonal derangements, including elevated levels of atrial natriuretic peptide, renin, aldosterone and norepinephrine, have been demonstrated in patients with congenital heart disease, persisting many years after surgical repair of cardiac defects [21–24]. Similarly, global impairment of autonomic nervous system regulation is present and persists late after repair of a number of congenital heart disease lesions, including Tetralogy of Fallot and Fontan-type operations [25, 26]. Given the central role of these neural and hormonal mechanisms in renal physiology, including blood flow regulation, intraglomerular hemodynamic control, and tubular function, they have significant potential to contribute to the evolution of CKD in congenital heart disease.

Chronic cardiorenal syndrome (also called Type 2) refers to clinical condition where chronic abnormalities of cardiac function cause progressive kidney disease. Multiple episodes of acute cardiac decompensation in patients with congenital heart disease could result in the onset or progression of kidney disease through mechanisms discussed above. In addition, podocyte injury (albuminuria and decreased glomerular nephrin and podocin mRNA has been shown in a model of chronic cardiac volume overload [27]. In humans, there is also direct evidence of podocyte injury (urinary podocyte loss) in acute decompensated Type 2 cardiorenal syndrome without overt precipitating factors [28]. The presence of such podocyte loss in chronic cardiorenal syndrome and its role in the development and progression of CKD need to be further investigated.

The impact of the intrinsic, pathophysiologic changes discussed above, and summarized in Fig. 1, has likely changed over time, as surgical timing, methods of repair, and heart failure management change over time. However many of these factors are not specifically modifiable. This is in contrast to extrinsic factors related to the medical management of patients with congenital heart disease, several of which may be modifiable, summarized in Fig. 1 and discussed following.

### Cardiopulmonary bypass is associated with acute kidney injury (AKI)

In addition to the pathophysiological changes in congenital heart disease which can impact kidney structure and function as described above, children with congenital heart disease are exposed to cardiac surgery with cardiopulmonary bypass, and resulting AKI. AKI occurs in 20 to 30 % of children undergoing cardiac surgery with cardiopulmonary bypass for congenital heart lesions; in neonates, this rate can be as high as 60 %. [29–34]. Although there are multiple definitions of AKI, all based on serum creatinine, that can lead to variation in incidence or prevalence of this condition, the consistent finding is that AKI is common after cardiac surgery. Many children with congenital heart disease must have multiple cardiac surgeries, leading to higher cumulative AKI risk. Cardiac surgery associated AKI is not a new phenomenon, although it is being given more attention recently as it has become clear that it is associated with negative short term outcomes, including increased illness-severity adjusted ventilation time, intensive care stay, and hospital stay [29–32]. AKI was recognized (at least in the literature) in the 1960s [35] as a complication of cardiac surgery, and the paradigm over time of organ injury after heart surgery has largely been based on ischemia-reperfusion pathophysiology. Animal models provide some evidence that AKI sustained in this way can have long-term kidney consequences; after ischemia-reperfusion injury, cellular damage of the endothelial and tubular cells within the kidney leads to endothelial cell differentiation, impaired regenerative capacity, vascular dropout and tubulointerstitial fibrosis [36–38]. Despite the significant time that has passed since the first open heart surgery on a “blue baby” in 1944, there has been a knowledge gap in understanding how AKI following cardiac surgery impacts the kidney long-term. As summarized further in this review, current evidence is beginning to close this gap.

### Drugs used in children with congenital heart disease are often nephrotoxic

Several drugs used frequently in the setting of congenital heart disease have known nephrotoxicity. One such class of drugs are angiotensin converting enzyme inhibitors, which are used to control blood pressure, balance single-ventricle circulations, and prevent pathologic ventricular remodeling. A recent retrospective study of 206 hospitalized neonates with congenital heart disease demonstrated a significant decrease in renal function (estimated creatinine clearance) while they were receiving angiotensin converting enzyme inhibitor; 42 % of patients had AKI (with 70 % of these being classified as renal failure by modified pRIFLE criteria) [39]. As the authors acknowledge, this study was limited by its lack of control group, the confounding potential of interventions such as cardiopulmonary bypass and nephrotoxic contrast exposure, and progression of heart disease/failure as a possible explanation of their findings (vs. the effect of drug). Despite these limitations and because no substantial body of evidence supports angiotensin converting enzyme inhibitor use as beneficial in this population, the authors suggested limiting these nephrotoxic medication whenever possible. An additional retrospective review of 319 young children however demonstrated no increase risk of AKI with angiotensin converting enzyme inhibitor, even in combination with furosemide [40]. In the context of this review, in the absence of stronger evidence including a randomized trial, it is difficult to generalize and apply this statement to all patients with congenital heart disease. However cautious use of angiotensin converting enzyme inhibitors with a goal of minimizing AKI is a reasonable, potentially beneficial, practice. The long-term renal effects of angiotensin converting enzyme inhibitors or AKI associated with their use in patients with congenital heart disease is unknown.

Other drugs with effects on the kidney used commonly in congenital heart disease which are possible modifiable risk factors for AKI (and therefore potentially CKD as discussed below) are non-steroidal anti-inflammatories and diuretics. Inappropriate use of loop diuretics may exacerbate renal hypoperfusion through vasodilatation and excessive diuresis resulting in worse outcomes. Moffett et al. demonstrated that the co-administration of furosemide is an independent risk factor for AKI in children receiving angiotensin converting enzyme inhibitor after cardiac surgery [41]. In a recent systematic review evaluating loop diuretic use in established AKI, very low quality evidence showed that there may be an increase in mortality and need for dialysis with loop diuretics, however this is not specific to the congenital heart disease patient and the uncertainty of these effects was too large to make clear conclusions about clinical harm [42]. There is nothing known regarding the long-term use of furosemide in congenital heart disease patients as it related to long-term renal outcome. Non-steroidal anti-inflammatories (eg. ketoraloc) are used for analgesia in children that undergo congenital heart repairs. Cyclo-oxygenase metabolites and prostaglandins play a significant role in regulating renal vascular tone and kidney function; pharmacological inhibition of these mediators by non-steroidal anti-inflammatories could produce deleterious effects leading to AKI. Several small, retrospective studies have demonstrated that ketorolac after congenital heart surgery is not been associated with a measurable difference in renal function in infants [43, 44] or after low-risk cardiac procedures in older children [45]. A randomized trial has shown that short-term use of ketorolac after congenital heart surgery does not result in AKI, however AKI was not the primary outcome of the study and it was under-powered to detect this effect [46]. Hence, definitive evidence regarding the role of this commonly used medication in AKI in people with congenital heart disease is lacking.

### There is a transition from AKI to CKD

In adults, there is evidence that AKI is associated with the development or progression of CKD, in both cardiac surgery and non-cardiac populations. A recent meta-analysis has demonstrated that AKI in adults is associated with a 9-fold increased risk of developing CKD, and a 3-fold increased risk of developing end-stage kidney disease [47]. A more specific meta-analysis of AKI associated with cardiopulmonary bypass in adults, evaluating prognosis, found increased long term mortality and stroke, but renal outcomes were not measured in a significant proportion of the studies [48]. In children, there is some retrospective data demonstrating AKI as a risk factor for CKD; Mammen et al. evaluated 126 critically ill children with AKI and demonstrated that at 1-3 years of follow-up, 10 % of children developed CKD (defined as estimated glomerular filtration rate (eGFR) < 60 mL/min/1.73 m^2^ or persistent albuminuria) [49]. Forty-seven percent of children with a history of AKI were considered at risk of CKD (defined as eGFR of 60-90 mL/min/1.73 m^2^, hyperfiltration (eGFR >150 mL/min/1.73 m^2^), or hypertension). There are large, multicenter research studies currently underway, including our work, with primary aims of determining the risk of developing future CKD associated with AKI [50].

As noted above, in the general population, AKI events have been associated quite strongly with CKD during follow up. Given that patients with congenital heart disease have a significant number of other factors with the potential to modify both renal injury and recovery (i.e. alterations in cardiac output, neurohormonal/ autonomic activation, and renal perfusion) they may have different, potentially increased, susceptibility to this AKI-to-CKD transition (Fig. 1). The AKI-to-CKD transition is critical to understand in patients with congenital heart disease, as interventions to reduce AKI occurrence and complications after cardiac surgery could have impact on long-term renal outcomes in this cohort. Minimizing nephrotoxic drug exposure whenever possible is a rational approach to minimize AKI. Other interventions such as early peritoneal dialysis catheter placement [51] and renoprotective pharmacological agents need randomized studies powered to detect clinically relevant differences in outcome, first short-term outcome and then long-term outcome. For the most part, these studies do not currently exist [52]. Integral to understanding the AKI to CKD transition in patients with congenital heart disease, there needs to be accurate diagnosis of AKI and early risk stratification of those who will go on to develop chronic disease (for targeting follow up). Although there has been a lot of work done in the last 10 years in biomarker discovery for the diagnosis of AKI in a variety of critical care populations including congenital heart disease, a major knowledge gap exists in biomarker use for identifying processes leading to CKD.

### CKD has been clearly demonstrated in patients with congenital heart disease

A number of older, small studies have suggested that renal dysfunction exists in adults with congenital heart disease. Aperia et al. demonstrated decreased GFR (by inulin clearance) in 5 out of 10 adults with Tetralogy of Fallot, a mean of 20 years post Blalock-Taussig shunt [11]; mean GFR in these adults was 80 mls/min/1.73 m^2^. These results are in agreement with a small study by Dittrich et al. demonstrating that decreased GFR (by 24 h urinary creatinine clearance) was a common finding in long-standing cyanotic congenital heart disease [53]. In another cohort of young adults (n = 83), Flanagan et al. demonstrated proteinuria, defined as ≥2+ (100 mg/dL) on spot urinalysis, in one-third of cyanotic congenital heart disease patients. Risk of proteinuria was significantly higher than in an acyanotic control group with surgically corrected cyanotic congenital heart disease (Tetralogy of Fallot or transposition of the great vessels) [54]. Reasons for differences between these groups were not explored in detail, particularly in relation to their early childhood course, prior surgical procedures, or concomitant medication use; however as expected, many patients with cyanotic congenital heart disease had more complex heart disease. The hematocrit was not significantly different. Chronic glomerular injury as a prominent feature of cyanotic congenital heart disease has similarly been suggested by additional data demonstrating both an elevated albumin/creatinine ratio and an elevated protein/creatinine ratio in 38 % (n = 26) of long-standing cyanotic congenital heart disease [53].

In addition to glomerular abnormalities as a prominent feature of the “nephropathy” associated with congenital heart disease, studies have also shown changes in renal tubular function. In a small study of 43 children with cyanotic and acyanotic congenital heart disease, Agras et al. found a significant increase in the fractional excretion of sodium and N-acetyl-B-D-glucosaminidase (used as a marker of proximal tubular damage) in children with cyanotic congenital heart disease [55]. These markers of proximal tubular dysfunction and injury were also elevated in non-cyanotic congenital heart disease relative to controls, although to a lesser extent than in cyanotic congenital heart disease. A more recent study of 58 children with congenital heart disease (with healthy matched controls) confirmed similar findings [56]. Of note, in both of these studies, the majority of children in this study were in lower risk congenital heart surgery classes (by Risk Adjustment for Congenital Heart Surgery 1), the duration of follow-up was not specified, and it was not clear if urine evaluations occurred before or after cardiac repair.

Recent data from a large, well designed study confirms the presence of CKD in patients with congenital heart disease [57]. In 1102 adults with congenital heart disease (both cyanotic and non-cyanotic), half met GFR criteria for a diagnosis of CKD (41 % had an eGFR (by modification of diet in renal disease) between 60 and 89 ml/min/1.73 m^2^ and 9 % had a GFR < 60 ml/min/1.73 m^2^); the risk of renal impairment was significantly higher in cyanotic patients [57]. When compared to the general population, the prevalence of significant renal impairment in this study was 18-fold higher in non-cyanotic and 35-fold higher in cyanotic congenital heart disease patients [57]. The study sample was patients seen at a tertiary care referral center for congenital heart disease with the first creatinine at referral used to estimate GFR and potentially including patients with acute cardiac decompensation, hence may not represent the general population of congenital heart disease. This study importantly demonstrated that it is not just those patients with cyanotic congenital heart disease that are at increased risk of CKD but also non-cyanotic, and changes occur fairly early in adulthood with a mean age at assessment of 36 ± 14 years. Although CKD has been clearly demonstrated in patients with congenital heart disease, little else is known about its characteristics. This cohort has a number of factors that could influence the development or progression of CKD, including common interventions for heart failure such as angiotensin converting enzyme inhibitors [58].

A number of investigators have tried to sort out the role of AKI after cardiac surgery in the development of CKD in patients with congenital heart disease. In one of the earliest studies to describe longer-term renal outcomes, Shaw et al. report various abnormalities in renal function in 6 out of 11 children with congenital heart disease 1 to 5 years after severe AKI (requiring dialysis). Abnormalities included not only low measured GFR (using radioisotope) in keeping with current CKD definition, but also elevated GFR reflecting hyperfiltration, a marker of early renal damage. Additional abnormalities were those of tubular proteinuria and urine concentrating abnormalities [59]. Another more recent, but also small (n = 25), study reported no features of CKD in surviving children treated with peritoneal dialysis after surgical correction of congenital heart anomalies at 5 years median follow up. Of note, however, hyperfiltration by eGFR was again common in these children (36 %) and the investigators did not measure albumin/creatinine ratio, an important measurement for current CKD definitions [60].

### CKD in patients with congenital heart disease contributes to negative health outcomes

Although CKD contributes significantly to increase the risk of cardiovascular events and mortality in the general population [61], there is not yet a clear understanding of the impact of CKD in patients with congenital heart disease. Young adults with congenital heart disease who have decreased GFR have lower survival than those with normal GFR [57]. This is not simply because they have lower heart function; there is a clear additional effect of renal impairment over that of functional class and systemic ventricular function [57]. A population-based study in congenital heart disease patients surviving to > 65 years demonstrated that one of the most powerful predictors of mortality was CKD; the mortality risk associated with CKD was larger than that associated with cancer, heart failure, myocardial infarction, or diabetes [62]. A weakness in this data was ascertainment of CKD by ICD-9 coding (a method which was not rigorously validated) and hence, a very low prevalence of CKD. The mechanism for increased cardiovascular disease in patients with CKD appears to be due in part to an increased burden of cardiovascular risk factors and to the impact of accumulation of these risk factors over time.

Children with CKD have subclinical manifestations of vascular disease, including atherosclerosis with intimal plaque, multi-vessel calcification and arteriosclerosis [63]. Young adults with childhood onset CKD have increased coronary artery calcification and increased carotid intima-media thickness [63, 64]. Endothelial dysfunction is present in CKD, which is associated with hypertension, left ventricle hypertrophy, and increased cardiovascular disease events such as myocardial infarction [65–67]. The extent of vascular change is associated with the number of risk factors, their intensity, and exposure duration [68–70]. Thus, CKD-associated cardiovascular disease pathogenesis in children appears to begin early in life with exposure to the atherogenic milieu of CKD, speaking to the potential importance of early CKD detection and identifying risk factors of child CKD development. Given the large negative impact of CKD on health outcomes in the general population and the child’s potentially long life time to accumulate risk, CKD development in children with congenital heart disease could place them at high risk for future cardiovascular disease.

## Conclusions

Patients with congenital heart disease should be recognized as a population at risk of developing CKD. The pathophysiology in congenital heart disease can lead to changes in structure and function of the kidney. In addition, the presence of congenital heart disease concomitantly places patients at risk of exposure to factors that cause AKI, including cardiopulmonary bypass and nephrotoxins. Although limited, the current epidemiological evidence suggests that renal dysfunction and CKD occur in patients with congenital heart disease with higher frequency than the general population and are detectable early in follow-up (i.e. during childhood). Despite a relatively young age, the best evidence suggests that approximately 30 to 50 % of adult patients with congenital heart disease have significantly impaired renal function. The risk of CKD is higher with cyanotic congenital heart disease but it is also present with non-cyanotic congenital heart disease. Many questions still need to be answered, highlighted in this review: As many patients with congenital heart disease are now undergoing reparative (not just palliative) correction and earlier in life, will the risk of CKD stay the same over time? What extent does AKI play a role in CKD in patients with congenital heart disease? Does changing / modernization of intensive care management over time lead to a change in AKI incidence and hence change in CKD? How much CKD is related to modifiable drug exposure? How can we predict which patients with congenital heart disease will get CKD? What do we do to identify, prevent and follow cardiovascular risk in these patients?

Longer-term studies with strong methodology are almost non-existent, although they are in process and the results will fill significant knowledge gaps. There are currently no clear guidelines for clinicians in terms of renal assessment in the long term follow up after cardiac surgery in childhood, yet this population represents one in whom long-term primary and secondary prevention strategies to reduce CKD occurrence and CKD progression could be instituted to significantly change outcomes. Although there are no guidelines, the sum of the data suggests that patients with congenital heart disease should be followed from an early age for the development of CKD; there is an opportunity to mitigate CKD progression and negative renal outcomes by instituting universally accepted interventions including stringent blood pressure control and treatment of proteinuria. Ongoing generation, synthesis, and translation of evidence in this area are critically important, as the population of adult survivors of congenital heart disease expands.

## Abbreviations

AKI: Acute kidney injury
CKD: Chronic kidney disease
eGFR: Estimated glomerular filtration rate
GFR: Glomerular filtration rate

## References

[1] Marelli AJ, Mackie AS, Ionescu-Ittu R, Rahme E, Pilote L (2007). Congenital heart disease in the general population: changing prevalence and age distribution. Circulation.

[2] Billett J, Cowie MR, Gatzoulis MA, Vonder Muhll IF, Majeed A (2008). Comorbidity, healthcare utilisation and process of care measures in patients with congenital heart disease in the UK: cross-sectional, population-based study with case-control analysis. Heart.

[3] Mackie AS, Pilote L, Ionescu-Ittu R, Rahme E, Marelli AJ (2007). Health care resource utilization in adults with congenital heart disease. Am J Cardiol.

[4] Gorodetskaya I, Zenios S, McCulloch CE, Bostrom A, Hsu CY, Bindman AB (2005). Health-related quality of life and estimates of utility in chronic kidney disease. Kidney Int.

[5] Pichon A, Connes P, Quidu P, Marchant D, Brunet J, Levy BI (2012). Acetazolamide and chronic hypoxia: effects on haemorheology and pulmonary haemodynamics. Eur Respir J.

[6] Cordina RL, Celermajer DS (2010). Chronic cyanosis and vascular function: implications for patients with cyanotic congenital heart disease. Cardiol Young.

[7] DeFilippis AP, Law K, Curtin S, Eckman JR (2007). Blood is thicker than water: the management of hyperviscosity in adults with cyanotic heart disease. Cardiol Rev.

[8] Nashat FS, Portal RW (1933). The effects of changes in haematocrit on renal function. J Physiol.

[9] Passwell J, Orda S, Modan M, Shem-Tov A, Aladjem A, Boichis H (1976). Abnormal renal functions in cyanotic congential heart disease. Arch Dis Child.

[10] Burlet A, Drukker A, Guignard JP (1999). Renal function in cyanotic congenital heart disease. Nephron.

[11] Aperia A, Bjarke B, Broberger O, Thoren C (1974). Renal function in Fallot’s tetralogy. Acta Paediatr Scand.

[12] Shankland SJ, Ly H, Thai K, Scholey JW (1994). Increased glomerular capillary pressure alters glomerular cytokine expression. Circ Res.

[13] Blantz RC, Gabbai FB (1989). Glomerular hemodynamics in pathophysiologic conditions. Am J Hypertens.

[14] Spear GS (1960). Glomerular alterations in cyanotic congenital heart disease. Bull Johns Hopkins Hosp.

[15] Perloff JK, Latta H, Barsotti P (2000). Pathogenesis of the glomerular abnormality in cyanotic congenital heart disease. Am J Cardiol.

[16] Sahai A, Mei C, Schrier RW, Tannen RL (1999). Mechanisms of chronic hypoxia-induced renal cell growth. Kidney Int.

[17] Fine LG, Orphanides C, Norman JT (1998). Progressive renal disease: the chronic hypoxia hypothesis. Kidney Int Suppl.

[18] Sahai A, Mei C, Pattison TA, Tannen RL (1997). Chronic hypoxia induces proliferation of cultured mesangial cells: role of calcium and protein kinase C. Am J Physiol.

[19] Kim SB, Kang SA, Park JS, Lee JS, Hong CD (1996). Effects of hypoxia on the extracellular matrix production of cultured rat mesangial cells. Nephron.

[20] Truong LD, Farhood A, Tasby J, Gillum D (1992). Experimental chronic renal ischemia: morphologic and immunologic studies. Kidney Int.

[21] Ohuchi H, Takasugi H, Ohashi H, Yamada O, Watanabe K, Yagihara T (2004). Abnormalities of neurohormonal and cardiac autonomic nervous activities relate poorly to functional status in fontan patients. Circulation.

[22] Lang RE, Unger T, Ganten D, Weil J, Bidlingmaier F, Dohlemann D (1985). Alpha atrial natriuretic peptide concentrations in plasma of children with congenital heart and pulmonary diseases. Br Med J (Clin Res Ed).

[23] Ross RD, Daniels SR, Schwartz DC, Hannon DW, Shukla R, Kaplan S (1987). Plasma norepinephrine levels in infants and children with congestive heart failure. Am J Cardiol.

[24] Tulevski II, Groenink M, van Der Wall EE, van Veldhuisen DJ, Boomsma F, Stoker J (2001). Increased brain and atrial natriuretic peptides in patients with chronic right ventricular pressure overload: correlation between plasma neurohormones and right ventricular dysfunction. Heart.

[25] Davos CH, Davlouros PA, Wensel R, Francis D, Davies LC, Kilner PJ (2002). Global impairment of cardiac autonomic nervous activity late after repair of tetralogy of Fallot. Circulation.

[26] Davos CH, Francis DP, Leenarts MF, Yap SC, Li W, Davlouros PA (2003). Global impairment of cardiac autonomic nervous activity late after the Fontan operation. Circulation.

[27] Rafiq K, Noma T, Fujisawa Y, Ishihara Y, Arai Y, Nabi AH (2012). Renal sympathetic denervation suppresses de novo podocyte injury and albuminuria in rats with aortic regurgitation. Circulation.

[28] Le Jemtel TH, Rajapreyar I, Selby MG, Payne B, Barnidge DR, Milic N (2015). Direct evidence of podocyte damage in cardiorenal syndrome type 2: preliminary evidence. Cardiorenal Med.

[29] Toth R, Breuer T, Cserep Z, Lex D, Fazekas L, Sapi E (2012). Acute kidney injury is associated with higher morbidity and resource utilization in pediatric patients undergoing heart surgery. Ann Thorac Surg.

[30] Parikh CR, Devarajan P, Zappitelli M, Sint K, Thiessen-Philbrook H, Li S (2011). Postoperative Biomarkers Predict Acute Kidney Injury and Poor Outcomes after Pediatric Cardiac Surgery. J Am Soc Nephrol.

[31] Li S, Krawczeski CD, Zappitelli M, Devarajan P, Thiessen-Philbrook H, Coca SG (2011). Incidence, risk factors, and outcomes of acute kidney injury after pediatric cardiac surgery: a prospective multicenter study. Crit Care Med.

[32] Morgan CJ, Zappitelli M, Robertson CM, Alton GY, Sauve RS, Joffe AR (2013). Risk factors for and outcomes of acute kidney injury in neonates undergoing complex cardiac surgery. J Pediatr.

[33] Sethi SK, Goyal D, Yadav DK, Shukla U, Kajala PL, Gupta VK (2011). Predictors of acute kidney injury post-cardiopulmonary bypass in children. Clin Exp Nephrol.

[34] Aydin SI, Seiden HS, Blaufox AD, Parnell VA, Choudhury T, Punnoose A (2012). Acute kidney injury after surgery for congenital heart disease. Ann Thorac Surg.

[35] Ogg CS, Cameron JS (1969). Cardiovascular surgery and the kidney. Guys Hosp Rep.

[36] Sutton TA, Mang HE, Campos SB, Sandoval RM, Yoder MC, Molitoris BA (2003). Injury of the renal microvascular endothelium alters barrier function after ischemia. Am J Physiol Renal Physiol.

[37] Basile DP, Donohoe D, Roethe K, Osborn JL (2001). Renal ischemic injury results in permanent damage to peritubular capillaries and influences long-term function. Am J Physiol Renal Physiol.

[38] Basile DP, Friedrich JL, Spahic J, Knipe N, Mang H, Leonard EC (2011). Impaired endothelial proliferation and mesenchymal transition contribute to vascular rarefaction following acute kidney injury. Am J Physiol Renal Physiol.

[39] Lindle KA, Dinh K, Moffett BS, Kyle WB, Montgomery NM, Denfield SD (2014). Angiotensin-converting enzyme inhibitor nephrotoxicity in neonates with cardiac disease. Pediatr Cardiol.

[40] Phelps CM, Eshelman J, Cruz ED, Pan Z, Kaufman J (2012). Acute kidney injury after cardiac surgery in infants and children: evaluation of the role of angiotensin-converting enzyme inhibitors. Pediatr Cardiol.

[41] Moffett BS, Goldstein SL, Adusei M, Kuzin J, Mohan P, Mott AR (2011). Risk factors for postoperative acute kidney injury in pediatric cardiac surgery patients receiving angiotensin-converting enzyme inhibitors. Pediatr Crit Care Med.

[42] National Clinical Guideline Centre (UK). Acute kidney injury: prevention, detection and management up to the point of renal replacement therapy. London: Royal College of Physicians (UK); 2013 Aug National Institute for Health and Clinical Excellence: Guidance. 25340231

[43] Dawkins TN, Barclay CA, Gardiner RL, Krawczeski CD (2009). Safety of intravenous use of ketorolac in infants following cardiothoracic surgery. Cardiol Young.

[44] Moffett BS, Wann TI, Carberry KE, Mott AR (2006). Safety of ketorolac in neonates and infants after cardiac surgery. Paediatr Anaesth.

[45] Inoue M, Caldarone CA, Frndova H, Cox PN, Ito S, Taddio A (2009). Safety and efficacy of ketorolac in children after cardiac surgery. Intensive Care Med.

[46] Gupta A, Daggett C, Drant S, Rivero N, Lewis A (2004). Prospective randomized trial of ketorolac after congenital heart surgery. J Cardiothorac Vasc Anesth.

[47] Coca SG, Singanamala S, Parikh CR (2012). Chronic kidney disease after acute kidney injury: a systematic review and meta-analysis. Kidney Int.

[48] Pickering JW, James MT, Palmer SC (2015). Acute kidney injury and prognosis after cardiopulmonary bypass: a meta-analysis of cohort studies. Am J Kidney Dis.

[49] Mammen C, Al Abbas A, Skippen P, Nadel H, Levine D, Collet JP (2012). Long-term risk of CKD in children surviving episodes of acute kidney injury in the intensive care unit: a prospective cohort study. Am J Kidney Dis.

[50] Go AS, Parikh CR, Ikizler TA, Coca S, Siew ED, Chinchilli VM (2010). The assessment, serial evaluation, and subsequent sequelae of acute kidney injury (ASSESS-AKI) study: design and methods. BMC Nephrol.

[51] Madenci AL, Thiagarajan RR, Stoffan AP, Emani SM, Rajagopal SK, Weldon CB (2013). Characterizing peritoneal dialysis catheter use in pediatric patients after cardiac surgery. J Thorac Cardiovasc Surg.

[52] Patel NN, Rogers CA, Angelini GD, Murphy GJ (2011). Pharmacological therapies for the prevention of acute kidney injury following cardiac surgery: a systematic review. Heart Fail Rev.

[53] Dittrich S, Haas NA, Buhrer C, Muller C, Dahnert I, Lange PE (1998). Renal impairment in patients with long-standing cyanotic congenital heart disease. Acta Paediatr.

[54] Flanagan MF, Hourihan M, Keane JF (1991). Incidence of renal dysfunction in adults with cyanotic congenital heart disease. Am J Cardiol.

[55] Agras PI, Derbent M, Ozcay F, Baskin E, Turkoglu S, Aldemir D (2005). Effect of congenital heart disease on renal function in childhood. Nephron Physiol.

[56] Zheng J, Yao Y, Han L, Xiao Y (2013). Renal function and injury in infants and young children with congenital heart disease. Pediatr Nephrol.

[57] Dimopoulos K, Diller GP, Koltsida E, Pijuan-Domenech A, Papadopoulou SA, Babu-Narayan SV (2008). Prevalence, predictors, and prognostic value of renal dysfunction in adults with congenital heart disease. Circulation.

[58] Sommers C, Nagel BH, Neudorf U, Schmaltz AA (2005). Congestive heart failure in childhood. An epidemiologic study. Herz.

[59] Shaw NJ, Brocklebank JT, Dickinson DF, Wilson N, Walker DR (1991). Long-term outcome for children with acute renal failure following cardiac surgery. Int J Cardiol.

[60] Mel E, Davidovits M, Dagan O (2014). Long-term follow-up evaluation of renal function in patients treated with peritoneal dialysis after cardiac surgery for correction of congenital anomalies. J Thorac Cardiovasc Surg.

[61] Tonelli M, Wiebe N, Culleton B, House A, Rabbat C, Fok M (2006). Chronic kidney disease and mortality risk: a systematic review. J Am Soc Nephrol.

[62] Afilalo J, Therrien J, Pilote L, Ionescu-Ittu R, Martucci G, Marelli AJ (2011). Geriatric congenital heart disease: burden of disease and predictors of mortality. J Am Coll Cardiol.

[63] Kavey RE, Allada V, Daniels SR, Hayman LL, McCrindle BW, Newburger JW (2006). Cardiovascular risk reduction in high-risk pediatric patients: a scientific statement from the American Heart Association Expert Panel on Population and Prevention Science; the Councils on Cardiovascular Disease in the Young, Epidemiology and Prevention, Nutrition, Physical Activity and Metabolism, High Blood Pressure Research, Cardiovascular Nursing, and the Kidney in Heart Disease; and the Interdisciplinary Working Group on Quality of Care and Outcomes Research: endorsed by the American Academy of Pediatrics. Circulation.

[64] Oh J, Wunsch R, Turzer M, Bahner M, Raggi P, Querfeld U (2002). Advanced coronary and carotid arteriopathy in young adults with childhood-onset chronic renal failure. Circulation.

[65] London GM, Guerin AP, Marchais SJ, Metivier F, Pannier B, Adda H (2003). Arterial media calcification in end-stage renal disease: impact on all-cause and cardiovascular mortality. Nephrol Dial Transplant.

[66] Qunibi WY (2004). Consequences of hyperphosphatemia in patients with end-stage renal disease (ESRD). Kidney Int Suppl.

[67] Raggi P, Boulay A, Chasan-Taber S, Amin N, Dillon M, Burke SK (2002). Cardiac calcification in adult hemodialysis patients. A link between end-stage renal disease and cardiovascular disease?. J Am Coll Cardiol.

[68] Arnlov J, Evans JC, Meigs JB, Wang TJ, Fox CS, Levy D (2005). Low-grade albuminuria and incidence of cardiovascular disease events in nonhypertensive and nondiabetic individuals: the Framingham Heart Study. Circulation.

[69] Deckert T, Feldt-Rasmussen B, Borch-Johnsen K, Jensen T, Kofoed-Enevoldsen A (1989). Albuminuria reflects widespread vascular damage. The Steno hypothesis. Diabetologia.

[70] Atiyeh BA, Dabbagh SS, Gruskin AB (1996). Evaluation of renal function during childhood. Pediatr Rev.

